# The consistent differential expression of genetic pathways following exposure of an industrial *Pseudomonas aeruginosa* strain to preservatives and a laundry detergent formulation

**DOI:** 10.1093/femsle/fny062

**Published:** 2018-03-14

**Authors:** Angharad E Green, Alejandro Amézquita, Yvan Le Marc, Matthew J Bull, Thomas R Connor, Eshwar Mahenthiralingam

**Affiliations:** 1 Microbiomes, Microbes and Informatics Group (MMI), Cardiff School of Biosciences, Cardiff University, Cardiff CF10 3AX, UK; 2 Unilever, Safety and Environment Assurance Centre (SEAC), Bedfordshire MK44 1LQ, UK

**Keywords:** *Pseudomonas aeruginosa*, antimicrobial resistance, transcriptomic RNA-Seq analysis, benzisothiazolone, phenoxyethanol, MexPQ-OpmE efflux pump

## Abstract

*Pseudomonas aeruginosa* is a common contaminant associated with product recalls in the home and personal care industry. Preservation systems are used to prevent spoilage and protect consumers, but greater knowledge is needed of preservative resistance mechanisms used by *P. aeruginosa* contaminants. We aimed to identify genetic pathways associated with preservative exposure by using an industrial *P. aeruginosa* strain and implementing RNA-Seq to understand gene expression changes in response to industry relevant conditions. The consistent differential expression of five genetic pathways during exposure to multiple industrial growth conditions associated with benzisothiazolone (BIT) and phenoxyethanol (POE) preservatives, and a laundry detergent (LD) formulation, was observed. A MexPQ-OpmE Resistance Nodulation Division efflux pump system was commonly upregulated in response to POE, a combination of BIT and POE, and LD together with BIT. In response to all industry conditions, a putative sialic acid transporter and isoprenoid biosynthesis *gnyRDBHAL* operon demonstrated consistent upregulation. Two operons *phnBA* and *pqsEDCBA* involved in *Pseudomonas* quinolone signaling production and quorum-sensing were also consistently downregulated during exposure to all the industry conditions. The ability to identify consistently differentially expressed genetic pathways in *P. aeruginosa* can inform the development of future targeted preservation systems that maintain product safety and minimise resistance development.

## Teaser

Exposing an industrial *Pseudomonas aeruginosa* strain to multiple industry relevant conditions associated with the preservatives benzisothiazolone and phenoxyethanol, and a laundry detergent, results in the consistent differential expression of five genetic pathways.

## INTRODUCTION

The opportunistic pathogen *Pseudomonas aeruginosa* demonstrates proficiency at surviving in environments with minimal nutritional levels (Favero *et al.*^1971^), exhibits multiple metabolic capabilities (Stanier, Palleroni and Doudoroff 1966) and has a genome size range of ∼5.2–7.0 Mbp (Stover *et al.*^2000^). These key attributes allow the microorganism to grow and proliferate within a variety of environments. Due to its dominance as an opportunistic pathogen in health care settings and patient groups such as those with Cystic Fibrosis, research has mainly focused on the epidemiology, pathogenesis and treatment of clinical *P. aeruginosa* isolates. In comparison, there is an incomplete understanding of strains isolated from the natural environment and much less is known about isolates that are successful contaminants of non-sterile cosmetic and household products (Jimenez 2007). *P. aeruginosa* has been isolated from numerous harsh environmental conditions such as crude oil and petroleum hydrocarbons (Guo-liang *et al.*^2005^; Song *et al.*^2006^), the Antarctic (Villeret *et al.*^1997^), hazardous chemicals used in conventional leather-making (Lama *et al.*^2012^), disinfectants (Dantas *et al.*^2008^) and pharmaceutical and cosmetic products (Jimenez 2007).

Recent analysis of home and personal care (HPC) products recalled due to contamination identified *P. aeruginosa* as the most frequently isolated microorganism (35.48%) (Neza and Centini 2016). The presence of *P. aeruginosa* in HPC products is detrimental to industry as high levels of this bacterium has the potential to break down formulations (Smart and Spooner 1972), as well as posing a potential risk to those with weakened immune systems. *P. aeruginosa* has been found in cosmetics (Tan, Tuysuz and Otuk 2013), shampoo products (Neza and Centini 2016), contact lens solutions and eye cosmetic products, where it has been reported to cause a number of infections of the eye and surrounding tissues (Reid and Wood 1979; Blumenfeld *et al.*^2005^). This highlights the requirement to minimise the occurrence of HPC product contamination with this pathogen through the improvement of manufacturing practices, and with enhanced product preservation systems. In the HPC industry, preservative formulations are used to achieve stability and sterility of products during manufacture and after being sold to consumers (McDonnell and Russell 1999). Globally by weight, preservatives are the most common type of antimicrobial which microorganisms are exposed to, due to the high occurrence of HPC product usage (Gilbert and McBain 2003; Alvarez-Rivera *et al.*^2012^). Isothiazolinones and alcohol-based preservatives such as benzisothiazolone (BIT) and phenoxyethanol (POE) are widely used to prevent the proliferation of bacteria within aqueous-based HPC products (Brannan 1997; Alvarez-Rivera *et al.*^2012^). BIT is commonly found in household products such as liquid laundry detergents (LDs), washing-up liquids and all-purpose cleaners. The main mechanism of action for isothiazolinone preservatives is the oxidation of the thiol groups found frequently on bacterial enzymes and proteins; this results in the inhibition of growth and metabolic activity of microorganisms (Collier *et al.*^1990^; Williams 2007). In *P. aeruginosa*, the regulatory c-di-GMP phosphodiesterase RocR enzyme was found to be inhibited by a BIT derivative and a reduction in the ability to swarm was also observed (Zheng *et al.*^2016^). The preservative POE is frequently incorporated into personal care products which include shampoos, shower gels and body creams. The proposed mechanism of action of POE is the disruption of bacterial cell membranes, cell lysis and leakage of cellular protein material (Gilbert, Beveridge and Crone 1977; Fitzgerald, Davies and Russell 1992).

Since BIT and POE are widely used preservatives relevant to the HPC industry, expanding our understanding of how bacteria respond when exposed to them, and the potential resistance pathways utilised, is vital to ensure their sustained efficacy and to protect consumers. *P. aeruginosa* strains with tolerance to isothiazolinone preservatives have been identified from incidences of product contamination where spoilage has occurred (Chen *et al.*^2010^), suggesting the bacterium is able to overcome inadequate preservation strategies used in industry. This research aimed to understand *P. aeruginosa* in an industrial context by identifying key genetic pathways contributing to in-product preservative resistance with an industry relevant strain. After global transcriptomic analysis of the isolate was performed under HPC-relevant growth conditions, consistent differentially expressed genetic pathways were identified, which could form targets for improved preservative formulations.

## MATERIALS AND METHODS

Additional details can be found in the supplementary information (Supplementary information 1, Supporting Information)**.**

### Bacterial strains and growth conditions

A *P. aeruginosa* industrial strain, RW109, obtained from the Unilever culture collection, was used to investigate industrial antimicrobial resistance. The strain was stored at –80°C in Tryptone Soya Broth (TSB) containing 8% (vol/vol) dimethylsulfoxide (Fisher Scientific) and was routinely revived onto Trytone Soya Agar (TSA) (Oxoid Ltd), and incubated static for 24-h at 30°C. Overnight liquid cultures were prepared by inoculating 3 ml of TSB (Oxoid Ltd), with confluent growth from a 24-h plate culture, and incubated for 18-h shaking with aeration at 30°C. Throughout this study, an incubation temperature of 30°C was used to be more representative of industrial product manufacture and storage temperatures, but still enabling sufficient growth within an 18–24-h growth period.

### Preparation of preservative antimicrobial agents

The industrial preservatives BIT and POE were used as representative isothiazolinone and alcohol-based preservative categories, respectively. BIT was supplied as Proxel Gxl by Arch UK Biocides Ltd LONZA, with the I,2-benzisothiazolinon-3-one as the active ingredient. POE was supplied as Phenoxetol by Clariant GmbH Germany, with 2-Phenoxyethanol as the active ingredient. Stock concentrations of preservatives were calculated based on the manufacturer's designated active fraction of each agent present in the formulation; BIT was supplied as 20% active and POE as >99% active. Stocks were prepared in a % solution (vol/vol) in sterilised double-deionised water on day of use to 0.05% activity for BIT, and 2% activity for POE. Further dilutions were carried out in liquid growth media (vol/vol) to obtain the required test concentrations on the basis of % active preservative present. In addition, an unpreserved liquid laundry detergent (LD) formulation supplied by Unilever was also used and dilutions (vol/vol) were prepared in TSB growth media.

### Minimum inhibitory concentration testing of industrial preservatives

To obtain approximate Minimum Inhibitory Concentration (MIC) values for the preservatives BIT and POE, serial doubling dilutions of aqueous stock solutions were prepared in TSB to achieve final concentrations ranging from 0% to 0.05% for BIT and 0% to 2% for POE, and a microdilution broth assay was used.

### RNA-Seq exposure conditions

The RW109 strain was exposed to the preservatives BIT and POE individually at 50% of the MIC (test exposure conditions 1 and 2), BIT and POE in combination, both at 20% of the MIC (test exposure condition 3) and an unpreserved LD at a 1:100 dilution in combination with BIT at 20% of the MIC (test exposure condition 4). Exposure to TSB only was used as a control condition*.* Exposure was for 24-h at 30°C and Optical Density (OD) measurements were also taken at 15-min intervals; four biological replicates were obtained for the control and test exposure conditions (see supplementary information 1 for further information on how exposure experiments were set up). The time point of 24-h was chosen to allow RW109 to be in the presence of the industry relevant conditions for a substantial period of time before RNA was extracted. The mean Log_10_ OD value (450–580 nm) at 24-h for each test condition were also analysed by comparing the two means for the control and test exposure conditions using a paired two-sample t-test for means; Log_10_ OD value differences with a *P*-value of ≤0.05 were considered to be significant. To ensure there were viable cells after 24-h of exposure to the industry relevant conditions the number of colony forming units (CFU) were also determined at each point of harvest.

### Total RNA extraction, quantification, quality assessment and messenger RNA enrichment

Total RNA (toRNA) was extracted within 1 week of harvesting cells using the RiboPure RNA Purification Bacteria Kit (Ambion) according to the manufacturer's instructions. A Qubit fluorometer system with the broad range RNA kit (Invitrogen) was used to quantify the toRNA samples according to the manufacturer's instructions to insure the concentrations were >100 ng/μl. The quality of toRNA was assessed with a Bioanalyzer using the RNA 6000 Nano kit (Agilent Technologies Ltd), following the manufacturer's protocol. The toRNA samples were only used for further applications if the RNA Integrity Number was ≥8 and the ratio of 23S:16S rRNAs was ≥1.5. To enrich messenger RNA (mRNA) from the toRNA samples, the MICROBExpress bacterial mRNA enrichment kit (Ambion) was used according to the manufacturer's instructions.

### Complementary DNA library preparations and sequencing

Complementary DNA (cDNA) library preparations and sequencing were carried out in collaboration with the Genomics Research Hub at Cardiff School of Biosciences: https://www.cardiff.ac.uk/biosciences/research/technology-research-hubs/genomics-research.

### RNA-Seq data analysis and identification of differentially expressed genes

Bioinformatics analysis was carried out using a virtual machine, hosted by the Cloud Infrastructure for Microbial Bioinformatics (CLIMB) consortium (Connor *et al.*^2016^). Quality control and adaptor trimming of the sequencing sample raw reads was carried out using Trim Galore for paired-end reads (http://www.bioinformatics.babraham.ac.uk/projects/trim_galore/). The RNA-Seq reads were aligned to the RW109 complete genome sequence (obtained using Pacific Biosciences (PacBio) technology; see accession details below) via a Burrows-Wheeler Aligner transformation (BWA) with the BWA-MEM algorithm (v0.7.13-r1126) (Li 2013). The resulting Sequence Alignment/Map (SAM) files of aligned sequence reads were sorted into BAM files using the SAM Tools toolkit (v1.3) (Li *et al.*^2009^). The Python programme HTSeq-count (v0.6.0) (Anders, Pyl and Huber 2015) was used to count the number of sorted aligned RNA-Seq reads which map to the annotated gene features of the RW109 complete genome and differential gene expression was determined with the R Bioconductor programme DESeq2 (v1.14.1) (Love, Huber and Anders 2014). Differentially expressed genes (DEGs) were defined as exhibiting a log2-fold change of ≥1.5 as described previously (Sass *et al.*^2011^; Rushton *et al.*^2013^), along with an adjusted *P*-value of ≤0.05. Complete lists of the up- and downregulated genes for each test condition are provided as supplementary data files (RW109_Significantly_Up_Regulated_DEGs.xlsx and RW109_Significantly_Down_Regulated_DEGs.xlsx).

Amino acid sequences of DEGs in close proximity within the RW109 genome were further analysed to determine sequence homology with predicted functional pathways using the *Pseudomonas* database DIAMOND BLASTP tool (Buchfink, Xie and Huson 2015; Winsor *et al.*^2016^).

### Accession numbers

The raw RNA-Seq reads are available in the ArrayExpress database (http://www.ebi.ac.uk/arrayexpress) under accession number E-MTAB-6344. The complete *P. aeruginosa* RW109 genome is available at https://www.ebi.ac.uk/ena/data/view/GCA_900243355.1.

## RESULTS AND DISCUSSION

### Growth of RW109 when exposed to industry relevant conditions

**Figure 1.**
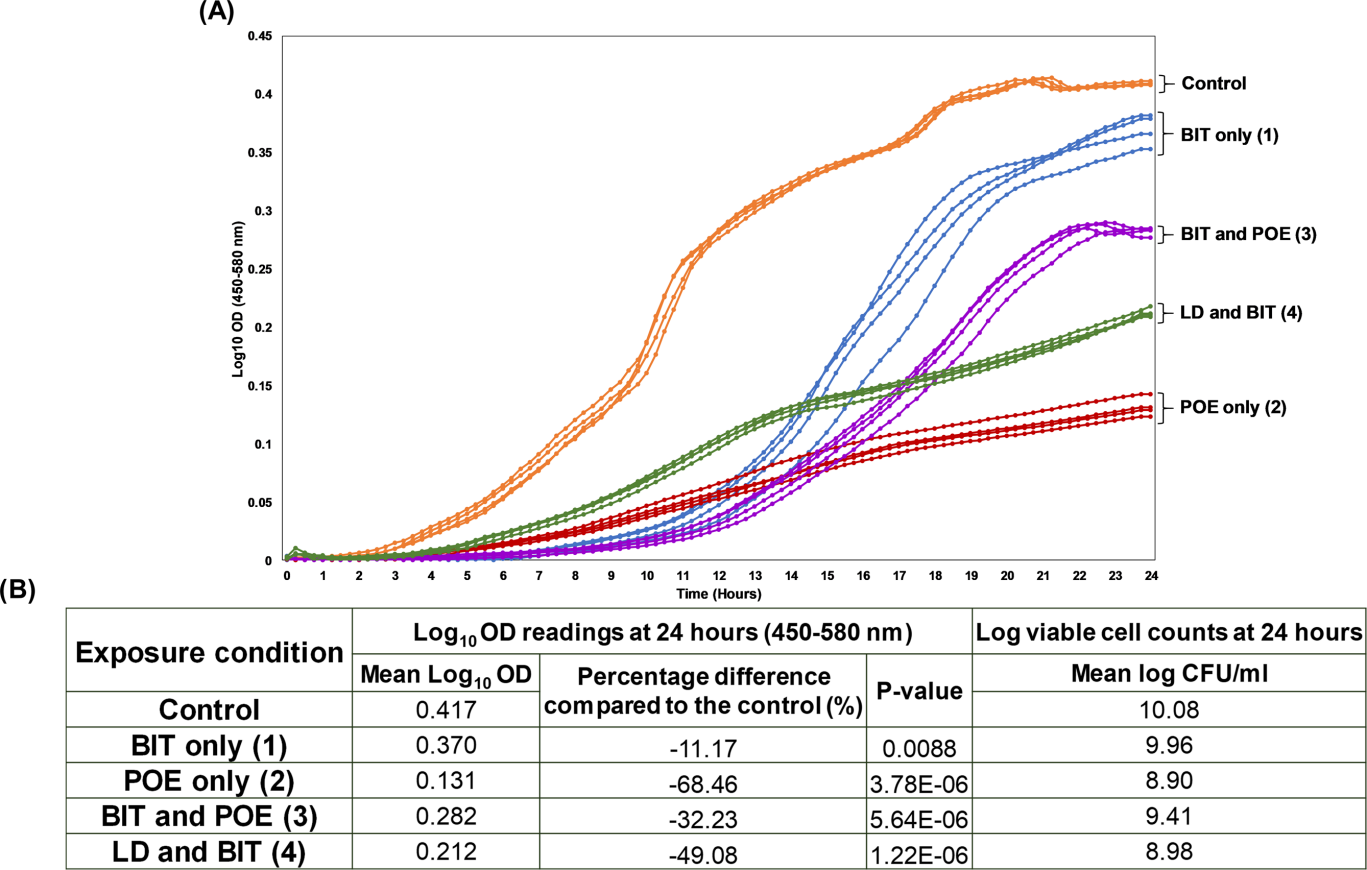
Growth dynamics of *P. aeruginosa* RW109 when exposed to the control and test conditions. The growth dynamics of RW109 when exposed to the control condition and test conditions (1–4) for 24-h at 30°C are represented by the line graph in (**A**). The four biological replicates of each exposure condition are plotted individually on the graph; the control is shown in orange, BIT at 50% of the MIC in blue, POE at 50% of the MIC in red, BIT and POE in combination in purple and LD and BIT in green. The mean Log10 OD (450–580 nm) at 24-h for each condition is shown in (**B**) along with the percentage difference in OD when test conditions were compared to the control condition. The mean viable cell count (log_10_ CFU/mL) after exposure to each condition for 24-h is also shown in (B).

*P. aeruginosa* RW109 had a higher tolerance of POE compared to BIT, with the MIC values calculated as 0.00938% active for BIT and 0.5% active for POE. When RW109 was grown in the presence of the control and test exposure conditions, the Log_10_ OD values were highly consistent during replicate exposure experiments (four replications; Fig. 1 A). BIT at 50% of the MIC (test condition 1) resulted in the smallest difference in mean Log_10_ OD when compared to the control with a percentage reduction of –11.17% observed (Fig. 1 B). POE at 50% of the MIC (test condition 2) had the greatest effect on RW109 growth in comparison to the control, with a –68.46% reduction in mean Log_10_ OD (Fig. 1 B). LD in combination with BIT (test condition 4) had the second greatest effect on OD with a –49.08% reduction observed, and this was followed by BIT and POE in combination (test condition 3), which resulted in an –32.23% OD reduction (Fig. 1 B). These results indicate that RW109 was able to grow when exposed to the industry relevant test exposure conditions over 24-h, but altered growth characteristics were observed in comparison to the control condition. For all exposure conditions, viable cells were present after 24-h (Fig. 1 B), indicating the RNA collected was from live bacteria.

### Overview of the RNA-Seq results

**Figure 2.**
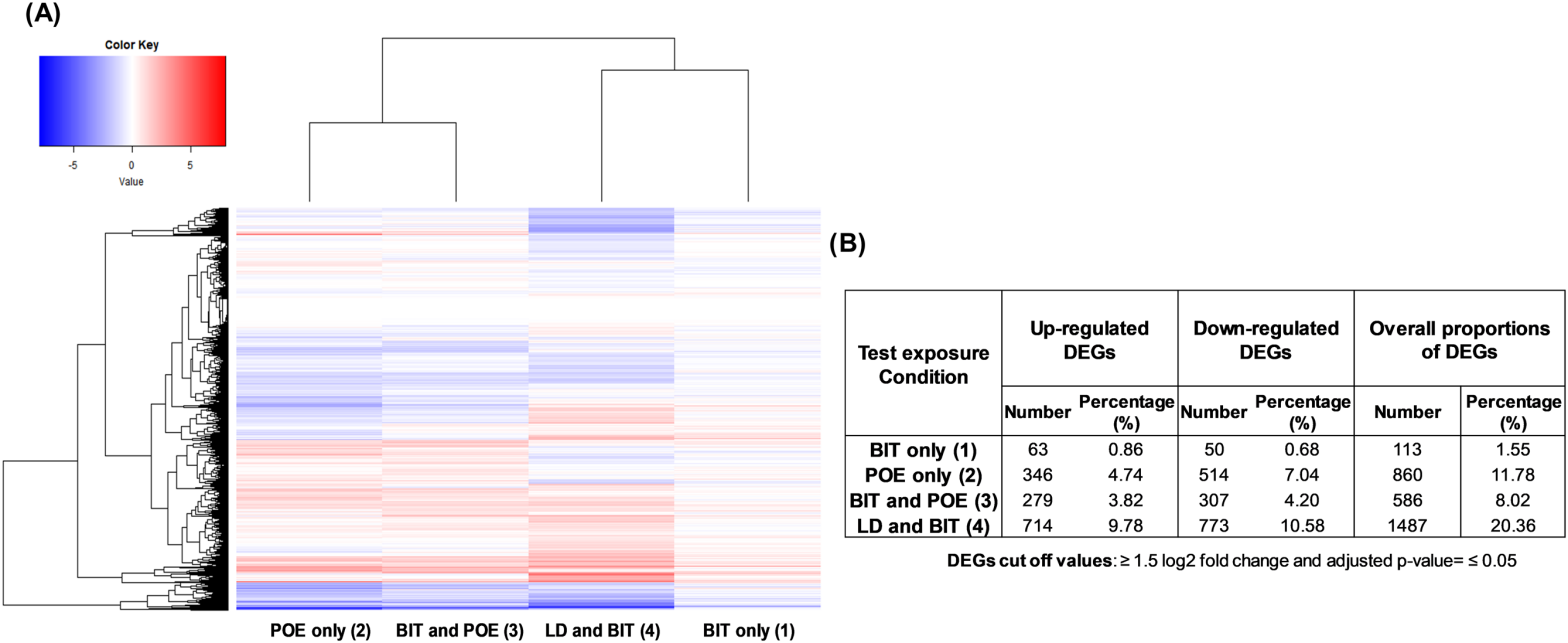
Overview of global gene expression in *P. aeruginosa* RW109 during exposure to industry relevant conditions. A heat map was generated (**A**) to show the overview of gene expression data for all the test exposure conditions when compared to the control. Red indicates genes which are upregulated and blue represents genes which are downregulated, and more intense the colours the greater the gene expression (see colour key). The top dendrogram displays the test exposure conditions which have been grouped together via hierarchical cluster analysis, and the dendrogram to the left of the heat map represents the gene clusters which are grouped according to their log2-fold change values across all the conditions. The number and percentage of genes which were differentially regulated for each test exposure condition are displayed in (**B**).

After sequencing, the resulting number of paired-end reads for all sample conditions ranged from 6.07E + 06 to 1.37E + 07. The reads were aligned to the sequenced RW109 genome, and a mean percentage number of 97.87% were found to align (range of 91.32%–99.03%). From these aligned reads, the mean percentage number which mapped to a gene feature was calculated as 98.56% (range of 96.74%–99.23%). Limited differential gene expression occurred when RW109 was exposed to the preservative BIT at 50% of the MIC (test condition 1); 1.55% of the genome demonstrated an expression change with 63 genes upregulated and 50 downregulated (Fig. 2). In response to POE only (test condition 2), 11.78% of the genome was differentially expressed with 346 genes upregulated and 514 downregulated (Fig. 2). An 8.02% gene expression change was observed when RW109 was exposed to BIT and POE in combination (test condition 3) with 279 genes upregulated and 307 downregulated (Fig. 2). Following exposure to LD in combination with BIT at 20% of the MIC (test condition 4), the greatest number of gene expression alterations were observed with 20.36% of the genome being differentially expressed, including 714 genes upregulated and 773 downregulated (Fig. 2). This indicates a large global gene expression effort was necessary for RW109 to grow in the presence of this HPC product in combination with BIT.

A key finding of the transcriptomic analysis was the consistent upregulation of three genetic pathways and the downregulation of two operons in response to the different test exposure conditions.

### Genetic pathways with consistent upregulation

**Figure 3.**
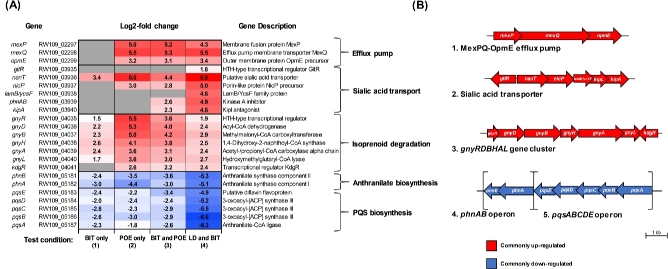
Common differentially expressed genetic pathways when *P. aeruginosa* RW109 was exposed to industry relevant conditions. The log2-fold changes (adjusted *P*-value of ≤0.05) of the five common differentially expressed genetic pathways following exposure to the four test conditions are shown in (**A**). The arrangements of these operons are displayed in (**B**). Each gene is drawn to the scale indicated by the 1 KB bar. For both panels A and B, red indicates genes which are upregulated and blue represents genes which are downregulated.

Three genetic pathways, efflux, sialic acid transport and isoprenoid degradation, were upregulated in response to industry relevant conditions, identifying them as common survival mechanisms used by *P. aeruginosa* RW109 when exposed to industrial preservatives and product formulations. The genes associated with an MexPQ-OpmE Resistance Nodulation Division (RND) efflux pump system were upregulated in response to POE at 50% of the MIC (test condition 2), BIT and POE in combination (test condition 3) and LD with BIT (test condition 4) (Fig. 3). Both MexP and MexQ were identified within the top 10 upregulated genes in response to test condition 3, and the MexQ gene was categorised within the top 10 upregulated genes after exposure to test conditions 2 and 4. This signifies the importance of the efflux genes during exposure to these industry relevant conditions. When exposed to BIT only, the efflux pump genes were not upregulated, suggesting it was not induced with this preservative when not in combination.

This efflux pump system is used by *P. aeruginosa* to export a variety of antibiotics such as fluoroquinolones, tetracycline, chloramphenicol and macrolides (Mima *et al.*^2005^). It has also been shown to function as a metal ion exporter, with *P. aeruginosa mexP* or *mexQ* mutants having increased susceptibility to copper (Thaden, Lory and Gardner 2010). The results from this study also indicate that the MexPQ-OpmE efflux system is as a resistance mechanism in response to POE only, POE and BIT, and LD in combination with BIT. The role of efflux as an important industrial preservative resistance mechanism was previously demonstrated with the upregulation of a *Burkholderia lata* RND-type efflux system in response to isothiazolone preservatives (Rushton *et al.*^2013^). Examples of *P. aeruginosa* efflux pump-mediated biocide resistance include the MexJK (Chuanchuen, Narasaki and Schweizer 2002) and MexAB-OprM (Schweizer 1998) efflux systems which export the biocide triclosan and the MexCD-OprJ efflux pump which is induced by disinfectants such as benzalkonium chloride, chlorhexidine and ethidium bromide (Morita *et al.*^2003^). This highlights the versatility of multidrug efflux pumps during exposure to agents other than antibiotics. The consistent upregulation of the *P. aeruginosa* MexPQ-OpmE RND efflux system genes in response to the industry relevant conditions used in this study emphasises the potential to target efflux pumps in preservation strategies. Chemicals that act as efflux pump inhibitors (Lomovskaya *et al.*^2001^) could be used in combination with industrial preservatives to potentiate their activity and enhance HPC preservation systems.

A putative sialic acid transporter gene (*nanT*, RW109_03936) demonstrated increased expression when RW109 was exposed to all four industry relevant conditions (Fig. 3). This gene was within the top 10 upregulated genes in response all four of the test exposure conditions, signifying its importance as a potential mechanism of survival in the presence of industry preservatives and HPC formulations. The sialic acid transporter encoding gene was identified within a predicted membrane transporter operon (RW109_03936-03940) (Fig. 3). In response to LD with the addition of BIT, all the genes within this operon were upregulated (Fig. 3), this suggests the importance of the genetic pathways for RW109 to survive the presence of this test condition. Sialic acids incorporate more than 50 naturally occurring nine-carbon amino sugars, which can be used by bacteria as sole carbon sources and are imported via sialic acid transporters (Vimr *et al.*^2004^). It is proposed that acquiring and assembling sialic acids on the surface mediates enhanced binding to host cells and facilitates the establishment of persistent infection (Greiner *et al.*^2004^; Khatua *et al.*^2010^). The *P. aeruginosa* RW109 strain may increase the expression of genes involved in the sialic acid transport system in order to uptake the molecule for enhanced survival in industrial conditions. The increased expression of this genetic pathway could also be used as an antimicrobial export mechanism of the preservative formulations and HPC products. The sialic acid transport system is clearly important during exposure to industry-related conditions and targeting its components in preservation strategies could be beneficial. A patent for an agent that specifically inhibits sialic acid transporters in numerous pathogens, including *P. aeruginosa*, has been filed and reports a reduction in bacterial growth upon exposure (Gibson, Munson and Post 2006). Agents such as this, which target common resistance mechanisms, could be incorporated into HPC preservative formulations to minimise proliferation of contaminants.

Genes within the isoprenoid degradation-associated *gnyRDBHAL* operon were also identified as commonly upregulated when exposed to the industrial test conditions (Fig. 3). The adjacent transcriptional regulator KdgR also had increased expression in response to conditions 2–4. The *gnyRDBHAL* gene cluster encodes enzymes that function in *P. aeruginosa* acyclic isoprenoid degradation (Diaz-Perez *et al.*^2004^). Increased transcript levels of the *gny* operon genes have also been observed during growth of *P. aeruginosa* in mucus when compared to cultivation in minimal media (Cattoir *et al.*^2012^). The frequent upregulation of these genes suggests a potential role of the *gnyRDBHAL* operon as a survival mechanism when RW109 was exposed to industrial conditions. This commonly differentially expressed pathway could also be targeted during the development of preservation systems due to its clear importance when exposed to industry relevant conditions.

### Genetic pathways with consistent downregulation

Two operons *phnAB* and *pqsABCDE* were frequently identified with decreased expression in response to all the industrial test conditions (Fig. 3). These operons were adjacent to each other within the RW109 genome and encode enzymes necessary for the biosynthesis of *Pseudomonas* quinolone signaling (PQS) molecules (Wade *et al.*^2005^). PQS are cell-to-cell signaling molecules involved in quorum sensing enabling the development of multicellular processes for survival purposes, such as biofilm formation (Diggle *et al.*^2007^). These molecules are generally produced in response to harsh environments and involve substantial investment of bacterial reserves (Häussler and Becker 2008). However, after prolonged exposure to growth rate limiting conditions such as preservative formulations and HPC products (Fig. 1), PQS production may not be sustainable. The *phnAB* and *pqsABCDE* operons were possibly beneficial during initial exposure to the industrial test conditions; however, after 24-h PQS biosynthesis may not have been viable for RW109. It is also proposed that under increased exogenous stresses, the overproduction of PQS becomes harmful to *P. aeruginosa* (D’Argenio *et al.*^2002^; Heurlier *et al.*^2005^).

Downregulation of the PQS genes in response to industrial preservatives may also be a mechanism, which allows the RW109 strain to persist in the presence of these antimicrobials and harsh conditions. Excessive PQS disrupts electron flow through the respiratory chain, which releases reactive oxygen species; this impairs membrane integrity inducing bacterial cell autolysis and the release of DNA (Hazan *et al.*^2016^). A *P. aeruginosa* mutant deficient in the *pqsA* gene was grown in the presence of the antibiotics ciprofloxacin, imipenem and gentamicin, and killing was found to be significantly delayed in comparison to the wild-type and a mutant which over produced PQS (Häussler and Becker 2008). The *pqsA* mutant also demonstrated an enhanced resistance when exposed to hydrogen peroxide in relation to the wild-type and the PQS overproducing mutant (Häussler and Becker 2008). As the industrial preservatives used in this study were membrane active agents, PQS biosynthesis may be detrimental to RW109 after prolonged exposure to the test conditions, resulting in the downregulation of the *phnAB* and *pqsABCDE* operons*.*

## CONCLUSIONS

This investigation identified genetic pathways in RW109 with functions in efflux, sialic acid transport, isoprenoid degradation and PQS biosynthesis, with consistent differential expression during exposure to different industry relevant conditions. These genetic pathways have previously been associated with bacterial stress response mechanisms, and this study indicates their importance when RW109 was exposed to preservatives and an LD formulation. The identified mechanisms could subsequently be targeted during the development of HPC preservation strategies to further enhance the safety of products used everyday by consumers.

## SUPPLEMENTARY DATA

Supplementary data are available at *FEMSLE* online.

## Supplementary Material

Supplemental data
