# Supplementary material for: The consistent differential expression of genetic pathways following exposure of an industrial Pseudomonas aeruginosa strain to preservatives and a laundry detergent formulation
Source: FEMS Microbiol Lett. 2018 Mar 14;365(9):fny062. doi: 10.1093/femsle/fny062 (PMC5905593; doi:10.1093/femsle/fny062)
Supplement: Supplemental data [file fny062_supp.zip › Supplementary Information 1.docx]

**Supplementary Information**

*MIC testing of industrial preservatives*

A micro-dilution broth assay was used to determine the MIC with the industrial RW109 strain with approximately 10^6^ cfu/ml of an overnight culture inoculated in wells of a micro-titre plate. The plates were incubated shaking (150 rpm) at 30°C, and after 24 hours the optical density (OD) at 600 nm of each well was recorded using the absorbance function of a Tecan Infinite® M200 PRO plate reader (Labtech International LTD, UK). MIC values were taken as the lowest concentration at which there was an 80% or more reduction in OD when compared to the growth in control wells containing TSB-only (Rose, 2009, Rushton *et al.*, 2013). Experiments were performed in triplicate using different starting cultures and preservative stock solutions to obtain biological replicates with each repeat having four technical replicates.

*RNA-Seq exposure conditions*

The exposure experiments were carried out using a Bioscreen C Microbiological Growth Analyser (Labsystems, Finland). Within a Bioscreen C microplate, four blank control wells contained 200 μl of TSB-only and were not inoculated with RW109 and four wells represented the control exposure conditions and contained 200 μl TSB-only inoculated with approximately 10^6^ cfu/ml of an RW109 overnight culture. Each test exposure condition was represented by four wells containing 200 μl of TSB supplemented with preservatives and/or laundry detergent and were inoculated with approximately 10^6^ cfu/ml of an RW109 overnight culture. Exposure was for 24 hours at 30°C and turbidity measurements were also taken at 15-minute intervals using a wide band filter (450-580 nm), after shaking the microplates for 10 seconds at an intermediate intensity. After exposure for 24 hours, cultures in each of the four technical replicate wells for the control and exposure conditions were promptly pooled into separate micro-centrifuge tubes, and immediately snap-cooled in an ethanol and dry ice bath, before centrifuging at 20,000 x g at 4ºC for 1 minute. The supernatant was removed and pellets were immediately snap-frozen and stored at -80ºC until required. Exposure experiments were repeated with different starting overnight cultures, and exposure condition preparations to obtain four biological replicates. The number of colony forming units (CFU) were also determined at the point of harvest for each exposure condition using the Miles and Misra surface viable plate count method (Miles *et al.*, 1938).

*Total RNA (toRNA) extraction, quantification and quality assessment*

Total RNA (toRNA) was carried out using the RiboPure™ RNA Purification Bacteria Kit (Ambion, Life Technologies Ltd, UK) according to the manufacturer’s instructions. After the extraction and wash steps, the RNA bound to a silica filter was eluted with 2 x 30 μl of elution buffer. Each sample was treated to remove any contaminating genomic DNA using the Ambion DNA-free™ reagents following the manufacturer’s instructions. An additional RNA precipitation step was incorporated for samples with low RNA concentrations (< 100 ng/μl) via addition of 0.1 volumes of 3 M sodium acetate, 5 μg of glycogen and 2.5 volumes of 100% ethanol. Mixtures were incubated for 30 minutes at -80°C, centrifuged at ≥12,000 x g for 30 minutes at 4°C, and RNA pellets washed twice with 70% ethanol and re-suspended in 17 μl of the elution buffer from the RiboPure™ RNA Purification Bacteria Kit and re-quantified. Following Bioanalyzer assessments, any samples with low quality were discarded and the exposure to the relevant condition and toRNA extraction was repeated.

*Messenger RNA (mRNA) enrichment, quantification and quality assessment*

The toRNA Samples were added to the MICROBExpress™ bacterial mRNA enrichment kit (Ambion) within a concentration range of 1.5 – 3 μg in a maximum volume of 15 μl. The resulting enriched mRNA pellets were re-suspended in 10 μl of nuclease free water and quantified using the Qubit™ fluorometer system, with the broad range RNA kit to ensure the concentration was greater than 2 ng/μl for input into the complementary DNA (cDNA) library preparation kit. The Bioanalyzer with the RNA 6000 Nano kit was used to evaluate the depletion of the16S and 23S rRNA peaks by comparing RNA samples before and after mRNA enrichment.

*cDNA library preparations and sequencing*

The Illumina® TruSeq® Stranded mRNA Sample Preparation Kit was used to prepare sequencing libraries with the low sample protocol. The manufacturer’s instructions describing the use of the protocol with previously isolated mRNA was followed; 5 μl of enriched mRNA within the Illumina recommended concentration range of 10 – 400 ng was added to 13 μl of the fragment, prime, finish mix to prepare sequencing libraries for each sample. Sample library concentrations were checked with the Qubit™ fluorometer system using the broad range DNA kit (Invitrogen) and were between the ranges of 20.0 – 83.3 ng/μl.

The fragment size of each library was evaluated using the Agilent Tape-System 2200, with the standard D1000 Screen-Tape and sizes varied between 290-320 bp. The libraries were then normalised to approximately 2 nM and pooled in equimolar concentrations. Pooled libraries were checked with the Qubit™ fluorometer, using the broad range DNA kit and the Agilent Tape-System 2200, with the High Sensitivity D100 Screen-Tape. The Illumina NextSeq-500 was used to carry out the sequencing and following the manufacturer’s instructions, pooled cDNA libraries were loaded into a mid-output cartridge to produce paired-end reads of 75 bp in length.

*RNA-Seq data analysis and identification of differentially expressed genes (DEGs)*

Trim Galore default settings were used to remove short low quality reads (< 20 bp), predict and trim Illumina adaptor sequences, and eliminate poor-quality bases from the sequences (< Q20). FastQC confirmed that the resulting trimmed reads were of sufficient quality for subsequent analysis. A complete sequence of RW109, obtained using Pacific Biosciences (PacBio) technology was used for all subsequent bioinformatics analysis of the RNA-Seq reads. An index database of the RW109 nucleotide FASTA file was generated using the BWA index and the BWA-MEM commands with default settings to align the paired-end trimmed RNA-Seq reads to the RW109 genome. For HTSeq-count, the input was the sorted alignment BAM files and the RW109 General Feature Format (GFF) file. The resulting HTSeq-count matrix files were input into the Bioconductor programme DESeq2 in R to normalise the count data and determine differential gene expression between the control and test samples. The p-values were adjusted via the p.adjust function with the Benjamini and Hochberg (BH) method.

Heat map analysis was carried out using the heatmap.2 function from the R package gplots (v2.3.2). A distance matrix was generated with log2-fold changes using the hierarchical cluster analysis ‘hclust’ ‘dist’ functions to produce a dendrogram tree. The hierarchical clustered distance matrix was used as an input for the heatmap.2 function. Initial overview analysis of all log-fold changes regardless of the adjusted p-value, were displayed as a heat map.

*References*

Miles AA, Misra S and Irwin J. The estimation of the bactericidal power of the blood. *Epidemiol Infect* 1938;**38**:732-749.

Rose HL. *Antimicrobial resistance of CF pathogens; mechanisms of biocide resistance and action*. 2009; PhD Thesis, Cardiff University (United Kingdom).
